# Treatment Failure of Dihydroartemisinin/Piperaquine for *Plasmodium falciparum* Malaria, Vietnam

**DOI:** 10.3201/eid2304.161872

**Published:** 2017-04

**Authors:** Bui Quang Phuc, Charlotte Rasmussen, Tran Thanh Duong, Le Than Dong, Mai Anh Loi, Didier Ménard, Joel Tarning, Dorina Bustos, Pascal Ringwald, Gawrie Loku Galappaththy, Nguyen Quang Thieu

**Affiliations:** National Institute of Malariology, Parasitology, and Entomology, Hanoi, Vietnam (B.Q. Phuc, T.T. Duong, M.A. Loi, N.Q. Thieu);; World Health Organization, Geneva, Switzerland (C. Rasmussen, D. Bustos, P. Ringwald, G.L. Galappaththy);; Institute of Malariology, Parasitology, and Entomology, Ho Chi Minh, Vietnam (L.T. Dong);; Institut Pasteur, Phnom Penh, Cambodia (D. Ménard);; Mahidol University, Bangkok, Thailand (J. Tarning)

**Keywords:** drug resistance, malaria, *Plasmodium falciparum*, artemisinin, Vietnam, piperaquine, mefloquine, dihydroartemisinin/piperaquine, antimicrobial resistance, vector-borne infections

## Abstract

We conducted a study in Binh Phuoc, Vietnam, in 2015 on the therapeutic efficacy of dihydroartemisinin/piperaquine for *Plasmodium falciparum* malaria. A high number of treatment failures (14/40) was found, and piperaquine resistance in Vietnam was confirmed. A change in the malaria treatment policy for Vietnam is in process.

The high failure rate of artemisinin-based combination therapy in the treatment of uncomplicated *Plasmodium falciparum* malaria has been a growing concern in the Greater Mekong Subregion of Southeast Asia. High numbers of treatment failures were reported for dihydroartemisinin/piperaquine in Cambodia (*1*), but efficacy of this drug has remained high in Vietnam since its introduction in 2003. We investigated dihydroartemisinin/piperaquine efficacy in the treatment of uncomplicated *P. falciparum* malaria in Binh Phuoc Province, Vietnam, during August–December 2015. We looked for molecular markers of drug resistance and determined piperaquine blood levels in treated patients to assess if piperaquine resistance was present in Vietnam. This study was approved by the ethics boards of the Ministry of Health in Vietnam and the Western Pacific Regional Office of the World Health Organization. 

The National Institute of Malariology, Parasitology, and Entomology conducted this study as part of routine surveillance on drug efficacy following the 2009 World Health Organization protocol (*2*). After obtaining written consent, patients (age of inclusion, 2–60 years) were enrolled and given dihydroartemisinin/piperaquine (Pharbaco, Hanoi, Vietnam) at a target dosage of 2.4 mg/kg for dihydroartemisinin and 18 mg/kg for piperaquine once a day for 3 days. Patients with treatment failures were subsequently given quinine hydrochlorate (30 mg/kg/d) and doxycycline (3 mg/kg/d) for 7 days. Primary endpoint was adequate clinical and parasitologic response (ACPR) on day 42; PCR genotyping, comparing day 0 and day of failure samples, was used to distinguish recrudescence from reinfection with another strain (*2*). Dried blood spots were collected on day 0 and analyzed for mutations in the *K13* propeller domain (*3*), *Pfmdr1* copy number (*4*), and *Pfplasmepsin2* (*PfPM2*) copy number (*5*), which are markers associated with artemisinin, mefloquine, and piperaquine resistance, respectively. By using a previously established relationship between capillary whole blood and venous plasma, piperaquine plasma concentrations were calculated from blood spots collected day 7 (*6*). Sequencing was done by the Institut Pasteur in Cambodia, and the piperaquine blood levels were assessed by the Mahidol Oxford Tropical Medicine Research Unit in Thailand.

Forty-six patients with uncomplicated *P. falciparum* malaria were enrolled; 44 were followed until day 42, and 2 were lost to follow-up after day 14. Mean age of enrolled patients was 26.9 (range 14–53) years, and 93% (43/46) were male. Geometric mean parasitemia on day 0 was 17,759 (range 1,514–97,454)/μL.

On day 3, half (23/46) of patients were parasitemic. On day 42, a total of 65% (26/40, 95% CI 48.3%–79.4%) had an ACPR, and 35% (14/40) had recrudescence; 4 were withdrawn because they became reinfected.

Artemisinin resistance is defined as delayed parasite clearance and is associated with mutations in the *K13* propeller domain, the most prevalent being the C580Y mutation in the eastern Greater Mekong Subregion (*7*). *K13* analysis of 42 samples (4 were excluded because of uninterpretable results) from our study showed that 90.5% (38/42) were C580Y and 9.5% (4/42) were wild-type. This C580Y prevalence is higher than that reported in a previous study done in Binh Phuoc in 2014, in which 34.5% of samples had the C580Y mutation (B.Q. Phuc, unpub. data).

Analysis of *PfPM2* showed that 25/46 (54.3%) samples had multiple copies of the gene. Of the 42 samples with known *K13* types, 22 (52.4%) had both C580Y and *PfPM2* amplifications. The remaining 3 had unknown *K13* types. All 46 samples had a single copy of *Pfmdr1*, indicating that all parasites were sensitive to mefloquine (*4*).

The average day 7 piperaquine plasma concentration (n = 42) was 35.7 (range 11.1–71.0) ng/mL. In 57.1% (24/42) of patients, this concentration was at or above the cutoff value (30 ng/mL) associated with adequate piperaquine exposure (*1*). For patients with ACPRs, the average concentration was 36.9 (range 17.2–71.0) ng/mL, and 57.7% (15/26) were adequately exposed. For patients that had recrudescence, the average concentration was 39.5 (range 12.4–65.7) ng/mL, and 72.7% (8/11) were adequately exposed.

Of the 14 patients who experienced recrudescence, 10 had parasites with the C580Y mutation and *PfPM2* amplifications, 3 had parasites with the C580Y mutation only, and 1 had parasites with an unknown *K13* type and *PfPM2* amplifications. *K13* mutations (found during routine surveillance conducted over the last 5 years in Vietnam) alone did not lead to dihydroartemisinin/piperaquine failures. The association between the presence of molecular markers and recrudescence is confounded by various factors, including parasite load, immunity, and drug levels. Of the 3 patients who had recrudescence and were infected with *P. falciparum* without *PfPM2* amplifications, 2 had inadequate piperaquine levels. Of the 11 patients who had recrudescence and an infection with *P. falciparum* with *PfPM2* amplifications, 7 had adequate piperaquine levels. Low piperaquine blood levels, irrespective of the presence of *PfPM2* amplifications, might play a role in some treatment failures. Treatment failures in cases with *PfPM2* amplification–positive parasites and adequate piperaquine exposure support the presence of piperaquine resistance in Vietnam. 

Our results show that 1 *K13* mutation has become dominant and that piperaquine resistance is present in Vietnam. A change in the malaria treatment policy to treat with artesunate/mefloquine in Binh Phuoc Province is underway.
